# Testing the Static-99R as a Global Screen for Risk of Sex Crime Recidivism in a Norwegian Routine Sample

**DOI:** 10.1177/1079063220951194

**Published:** 2020-08-22

**Authors:** Ingeborg Jenssen Sandbukt, Torbjørn Skardhamar, Ragnar Kristoffersen, Christine Friestad

**Affiliations:** 1 Oslo University Hospital, Norway; 2 University of Oslo, Norway; 3 University College of Norwegian Correctional Service, Lillestrøm, Norway

**Keywords:** recidivism, risk assessment, sex offenses, static-99, static risk factors

## Abstract

The Static-99R has been recommended for use as a first global screen for sorting out sex-convicted persons who are in need of further risk assessment. This study investigated the Static-99R’s predictive validity based on a nonselected Norwegian sample (*n* = 858) of persons released from prison after having served a sex crime sentence. After a mean observation period of 2,183 days, 3.4% (*n* = 29) had recidivated to a new sex offense. A higher number of recidivists were found among those with higher Static-99R total scores. The predictive contribution from each of the ten Static-99R risk items was investigated using standard logistic regression, proportional hazard regression, and random forest classification algorithm. The overall results indicate that the Static-99R is relevant as a risk screen in a Norwegian context, providing similar results concerning predictive accuracy as previous studies.

## Introduction

About 300 men are released in Norway each year after having served prison sentences for sexual offenses (Statistics Norway, 2017). International research has repeatedly shown that recidivism rates are low among individuals convicted of sexual offenses, especially when it comes to new sex offenses (Hanson & Bussiere, 1998; Hanson et al., 2016; Hanson & Morton-Bourgon, 2004, 2005). In a Nordic study of recidivism (Graunbøl et al., 2010), 3% of individuals convicted of sexual offenses in Norway recidivated within 2 years after release from prison, none of them into new sex crimes. The criterion for recidivism in this study was a new sentence that had to be served in the correctional service. Longer follow-up periods naturally provide higher recidivism rates, as indicated by a 5-year sexual recidivism rate of 8% in Norway (Grünfeld et al., 1998). This corresponds closely to international figures, as demonstrated in a recent study involving 21 samples from eight different countries with a 5-year sexual recidivism rate of 9.8% (Hanson et al., 2016). In spite of generally low recidivism rates, the issue of rehabilitation of individuals convicted of sexual offenses continues to raise public concern, often initiated by heavily exposed single cases involving reoffending. Although single cases are of limited value in understanding overall general patterns, they may direct attention toward the important issue of within-group differences in risk among individuals convicted of sexual offenses. Identifying the highest risk categories is important to direct limited resources toward the groups where the potential gain of intensified intervention is highest (see Kahn et al., 2017). This issue is becoming increasingly relevant as the proportion of individuals convicted of sexual offenses is increasing among the prison population (Sturge, 2018). Data from the Norwegian National Prison Registry (NPR) show that the proportion of prisoners incarcerated for sexual offenses has risen from 5% to 20% over the past 20 years. In 2010, stricter punishment for several types of sexual offenses was introduced in Norway (Prop. 97 L, 2009-2010). NPR data show that the average prison sentence length for persons convicted of sexual offenses increased from 606 days in 2010 to 867 days in 2019. According to Statistics Norway (2018), 46% of all sex offense convictions in Norway in 2018 resulted in a prison sentence.

### Screening for Risk of Sex Crime Recidivism

The need to screen for risk among convicted offenders and adjust correctional interventions according to level of risk and need in each individual case lies at the heart of the risk-need-responsivity (RNR) model (Andrews & Bonta, 2010). Adherence to these principles is assumed to increase the effectiveness of correctional programs, while lack of adherence to one or more of the principles may potentially increase recidivism (Dowden & Andrews, 2004). These principles also apply to persons convicted of sex crimes (Hanson, Bourgon, Helmus et al., 2009). In practical terms, adhering to the RNR principles requires a systematic approach to risk screening and assessment, based on reliable and valid instruments covering both static and dynamic risk factors, as the latter are seen to add incrementally to predictive accuracy as well as inform the choice of treatment targets (cf. Brankley et al., 2019). Several instruments conform to these standards, but they generally tend to be time-consuming and require access to information that is normally unavailable to the correctional services at intake. In situations where time, staff, and available information are limited, several countries have implemented actuarial risk tools as part of routine practice to assist them in distinguishing between correctional clients who vary in the probability to reoffend. An actuarial approach to risk assessment implies decision making based on fixed and explicit rules of how to score a set of empirically derived risk factors and combine these into a total risk score representing a prognosis of a future event (reoffending), expressed in probabilistic terms (Helmus & Babchishin, 2016).

### Static-99R as a Risk Screen

The Static-99R, as well as its precursor, the Static-99, was developed in Canada (Hanson, Phenix & Helmus, 2009; Helmus et al., 2012) based on samples from diverse jurisdictions, including European and U.S. samples (Hanson et al., 2016). The Static-99R is among the most used and best validated actuarial tools for persons convicted of sex crimes. Its ability to rank offenders according to their relative risk for sexual recidivism has been robust across different settings and samples (Hanson & Morton-Bourgon, 2009). Static-99R has been recommended for use as a first global screen to sort out those in need of further risk assessment with more elaborate instruments (De Vogel et al., 2004). According to Phenix, Fernandez, Harris et al. (2016), “the information provided by Static-99R can be thought of as a baseline estimate of risk for new sexual charges and convictions” (p. 6).

Several studies have supported Static-99R’s predictive abilities in European countries (Craig et al., 2004; De Vogel et al., 2004; Eher et al., 2013; Gonçalves et al., 2020; Sjöstedt & Långström, 2001). Hanson et al. (2011) reviewed 63 Static-99 predicting studies involving 70 distinct samples and found that the predictive accuracy of Static-99 was significant in Canada, the United States, the United Kingdom, New Zealand, Australia, Belgium, Germany, Denmark, Holland, Switzerland, Sweden, Austria, and Japan. Their findings showed that the Static-99 worked particularly well in the United Kingdom, Australia, and New Zealand and reasonably well in Canada, the United States, and continental Europe, leading the authors to conclude that Static-99 can be used with confidence in any of these countries. A Dutch sample of convicted offenders (Smid et al., 2014) found that Static-99R and Static-2002R showed a slight but consistent advantage in predictive properties over seven other structured risk assessment instruments across outcome measures and follow-up periods.

Predictive validity is not necessarily transferable to jurisdictions different from where the instrument was developed (Duwe & Rocque, 2018) and thus needs to be reinvestigated whenever an instrument is considered for introduction in a new setting. It is particularly important to replicate the findings in nonselected (routine) samples from other sociocultural and legal backgrounds. According to Långström (2004), Static-99 should be used with caution in non-Western countries, as his results showed low predictive accuracy for offenders who were recent immigrants or from an ethnic minority relative to the majority population. Recent studies have attempted to evaluate the predictive validity of Static-99/R across ethnic groups, and several studies show the potentially moderating role of offender race/ethnicity in risk research (Babischin et al., 2012; Lee & Hanson, 2017; Leguizamo et al., 2017; Smallbone & Rallings, 2013; Varela et al., 2013). Schmidt et al. (2017) found that the prediction of future sex offenses among offenders in Germany who had immigrated from the Near East and North Africa was not possible with Static-99R.

Studies from a Scandinavian context have supported the validity of Static-99 as a useful risk screen. Sjöstedt and Långström (2001) tested the predictive accuracy of the Static-99 in a retrospective follow-up study of a nationwide Swedish cohort of released men. Bengtson (2008) investigated a sample of forensically evaluated individuals who had sexually offended in Denmark. Her results indicated moderate predictive accuracy of Static-99 among persons convicted of child sexual abuse, but poor predictive accuracy for men convicted of rape (contact offenses against persons aged 15 or older). However, as Bengtson’s study was based on a highly selected high-risk sample, representing only 7% of all men sentenced for sexual offenses in the study period, it is difficult to draw any general conclusions about the instrument’s general predictive validity.

### Purpose of the Current Study

The purpose of the current study is to investigate the predictive validity of the Static-99R, based on a nonselected routine sample of persons released from prison in Norway after having served a sex crime sentence.

## Method

### Participants

The study is based on quantitative data drawn from the Norwegian National Prison Registry (NPR), including personal data on persons currently or formerly fulfilling legal sanctions administered by the correctional service. A nationwide cohort was established consisting of all men principally convicted of a sex offense, sentenced to prison, and released from imprisonment within the 4-year period 2010 to 2014. Persons sentenced solely to suspended or conditional sentences, community sentences, fines, or other sanctions than imprisonment were excluded from our sample. “Sex offenses” included all offenses covered by Chapter 19^1^ of the Norwegian Penal Code. This cohort (*n* = 1,289) was subjected to a follow-up, starting at release and lasting for a maximum of 9.2 years (mean number of days = 2,183). The information needed to score Static-99R was obtainable for 858 men (66% of the total number of those released), who constituted the final sample. Those not scored (*n* = 431) contained a subgroup of men with only Category B offenses^2^ (*n* = 84), for whom the use of Static-99R is not recommended. The rest (*n* = 347) were excluded from the study because the NPR contained insufficient information for scoring purposes. The excluded cases did not differ in ways that would be expected to impact Static-99R results or the results more broadly (e.g., age, marital status, criminal history). Thus, the sample in this study may be considered representative of persons sentenced to prison for sexual offenses in Norway.

Risk assessment is not part of routine practice in the Norwegian Correctional Service, so the Static-99R scoring presented in this article was done solely for the research purpose in the current study.

### Measures

#### Baseline variables

Static-99R consists of 10 risk factors (Table 2). All risk factors were scored as absent or present (0–1), except for two weighted items, Item 1 “age at release from index sex offense” (scored 1, 0, −1, −3, with increasing age) and Item 5 “prior sex offenses” (scored from 0 to 3, based on combined scores for charges and convictions). A score of 1 (yes) on Item 3 (index nonsexual violence) required a separate conviction for a nonsexual violent offense at the same time as the person was convicted of their index offense (Phenix, Fernandex, Harris et al., 2016). The Static-99R total scores may range from −3 to 12, reflecting five risk levels: low risk (I), below average risk (II), average risk (III), above average risk (IVa), well above average risk (IVb).

In addition to the risk factors included in Static-99R, we also registered the meted sentence (in days), days actually spent imprisoned (excluding days spent on remand), and date of release from prison.

#### Outcome variable

Recidivism was operationalized as a new sentence to be executed by the correctional service, conditioned on the date of the new offense occurring after the date of release. Follow-up started at the day of release from prison and ended in July 2019, which means a maximum follow-up time of 9.2 years (*M* = 6.3, third quartile = 7.4).

### Procedures

Information necessary to score the variables included in this study was retrieved from the NPR, based on conditions prevailing at the time of release from prison. Thus, the sex crime sentence from which a person was released in the years 2010 to 2014 was counted as the index offense, although this for some might not have been the most *recent* sexual offense. The outcome variable was the first new unconditional sentence (prison or probation order) to be served in the correctional service after the first release in the years 2010 to 2014, irrespective of whether the new sentence had been served or not. In most cases, those who incurred a new prison sentence were not imprisoned again, usually because they had not been summoned yet within the observation period. In the data set of all released sex offenders from 2010 to 2014, 99% only appeared once. Data were manually scored by the first author, who is trained in Static-99R and who, at the time of scoring, was blind to the outcome (recidivism). To test the reliability of the scoring, 20 randomly selected cases were independently scored by a second rater (the last author), also trained in Static-99R. The results indicated moderate agreement between the raters as illustrated by Cohen’s κ of 0.49, for the risk levels. Intraclass correlation defined by absolute agreement using a two-way random effects model, indicated strong agreement between the raters for the raw scores (intraclass correlation coefficient [ICC] = .85). Areas of discrepancies between the raters were thoroughly discussed to improve scoring consistency and ensure adherence to the manual’s instructions.

The research project was approved by the Norwegian Centre for Research Data, as well as the relevant correctional agencies.

### Data Analyses

The predictive accuracy of the Static-99R was investigated by receiver operating characteristic (ROC) curve analyses (Mossman, 1994), as these are less affected by base rates. The predictive contribution from each of the 10 Static-99R risk items was then investigated using standard logistic regression as applied in the ROC analysis.

As the follow-up time varied, we also analyzed time-to-recidivism using proportional hazard regression models to check if taking timing of events into account affected the results. For the hazard models, we report Harrell’s C as an overall measure of the model’s capacity to discriminate between outcomes, similarly as area under the curve (AUC) for logistic regression. Such models have also been used in previous studies (see Hanson et al., 2013; Phenix, Helmus, & Hanson, 2016), thus giving the additional advantage of making these estimates comparable to these studies. However, as the majority of the recidivism happened within a couple of years (median = 1.2 years, third quartile = 2.4 years, maximum = 4.5 years), the substantive results are expected to be similar.

In addition to these regression models, we applied a classification technique from the field of machine learning: the *random forest algorithm*. This is an ensemble method based on classification trees, which enables us to directly predict recidivism and address classification accuracy. While this technique is often referred to as *a black-box technique* as it does not provide any parameter estimates, the predictive value of each variable is examined by calculating the so-called variable importance. Variable importance is the mean decrease in classification accuracy when each variable is shuffled to not contribute to the prediction (Berk, 2016). Thus, which variables that have high/low importance can be compared to which variables turn out to be important in the regression analyses.

When using the Static-99R as a risk screen for an intervention, one would decide on a cut-off on the score. The focus would typically be on the higher nominal risk levels which should be targeted for further assessment and individually adapted intervention. A total score of 4 to 5 is classified as “above average risk” (risk level IVa) and a total score of 6+ is classified as “well above average risk” (risk level IVb) (Phenix, Fernandez, Harris et al., 2016). Risk level IVa or higher thus seems reasonable as cut-off for additional assessment/intervention. However, the ROC analysis works on a continuous probability scale that is slightly more complicated than such a classification. Classification error can therefore also be addressed using this cut-off and compared with actual recidivism rates. Thus, the classification accuracy of Static-99R and random forest algorithm can be compared directly.

### Prediction Accuracy for New Data

The purpose of using logistic regression models in this setting is to apply them to new data, where we do not know the actual recidivism. When the accuracy is evaluated on the same data used in the estimation of the regression model, the accuracy is typically higher than if applied on new data, a phenomenon known as *overfitting*. A more reliable evaluation, therefore, is to estimate the model on a proportion of the data and calculate AUC on the remaining smaller subsample. Unless there is a very large data set, this is costly, as one does not utilize all the data in estimation. An alternative is to apply the related technique of *k*-fold cross-validation, which splits the data *k* parts, and the model is estimated repeatedly while evaluated on the remaining subsample. The results are then aggregated over *k* folds, giving a more realistic AUC that can be expected for new data (James et al., 2013). This typically leads to a lower, but more realistic, accuracy. In addition to AUC on full data, we also report fivefold, cross-validated AUC, as well as the relative fit measures log likelihood and Akaike information criterion (AIC).

## Results

Table 1 presents descriptive information about the sample.

**Table 1.** Descriptive Statistics of Men Released From a Sex Offense Prison Sentence in Norway From 2010 to 2014 (*N* = 858).

Variable	Frequency	%	*M* (*SD*)	Range
Age (at release)			39.5 (14.8)	18–89
18–34	392	46		
35–39	88	10		
40–59	286	33		
60 or older	92	11		
Number of previous sentences			0.7 (2.2)	0–33
None	655	76	—	—
One	81	9	—	—
2–4	83	10	—	—
5–10	31	4	—	—
>10	8	1	—	—
Sentenced prison days	—	—	511.0 (622.3)	14–5,479
Days incarcerated (excl. remand)	—	—	345.4 (446.8)	2–5,449
Observation period (days)	—	—	2,305.6 (482.4)	87–3,366

As seen from the Table 1, a large majority (76%) had no previous convictions. Fifteen percent had previously been charged for or convicted of sex offenses. The mean length of punishment meted out among those released was 1 year 5 months (511 days), while time served in prison was approximately 11 months, on average. The longest sentence meted out was 14.9 years (5449 days).

In Table 2, the current sample’s scores on each single Static-99R item are compared with the item scores from the Static-99R development samples (as presented in Tsao & Chu, 2019, Table 1). Some differences appear in terms of criminal history: the Norwegian sample has a less comprehensive criminal history (fewer prior sex offenses and sentencing dates, as well as less nonsexual violence), but a somewhat higher proportion with previous convictions for noncontact sex offenses. In the Norwegian sample, victims were mainly unrelated females, while few were complete strangers.

**Table 2.** Comparison of Sample Characteristics on Static-99R Single Items.

Items on Static-99R			Current sample (*n* = 858)		Sample retrieved from Tsao & Chu (2019; Table 1, *n* = 4,644)	
			*n*	%	*n*	%
Age at release (years)						
Aged 18–34.9			392	45.7	1,625	35.0
Aged 35–39.9			88	10.3	742	16.0
Aged 40–59.9			286	33.3	1,904	41.0
Aged 60 or older			92	10.7	372	8.0
Ever lived with a lover for at least 2 years						
Yes			595	69.3	3,333	72.4
No			263	30.7	1,273	27.6
Index nonsexual violence—any convictions						
No			751	87.5	3,441	74.1
Yes			104	12.1	1,203	25.9
Missing			3	0.3		
Prior nonsexual violence—any convictions						
No			704	82.1	3,234	69.6
Yes			128	14.9	1,410	30.4
Missing			26	3.0		
Prior sex offenses	Charges^a^	Convictions				
	0	0	719	83.8	3,425	73.8
	1,2	1	95	11.1	700	15.1
	3–5	2,3	27	3.1	316	6.8
	6+	4+	8	0.9	203	4.4
Missing			9	1.0		
Prior sentencing dates (excluding index)						
3 or less			747	87.1	3,264	70.3
4 or more			94	11.0	1,380	29.7
Missing			17	2.0		
Any convictions for noncontact sex offenses						
No			663	77.3	3,996	86.0
Yes			187	21.8	648	14.0
Missing			8	0.9		
Any unrelated victims						
No			120	14.0	1,548	33.3
Yes			735	85.7	3,096	66.7
Missing			3	0.3		
Any stranger victims						
No			687	80.1	3,454	74.4
Yes			157	18.3	1,190	25.6
Missing			14	1.6		
Any male victims						
No			746	86.9	3,858	83.1
Yes			100	11.7	786	16.9
Missing			12	1.4		

a The Norwegian correctional client registry only includes information on charges ending in a sentence to be served in the Correctional Service. Charges dropped or acquitted are not part of a person’s criminal record and could not be scored in the Norwegian sample.

**Figure 1.**
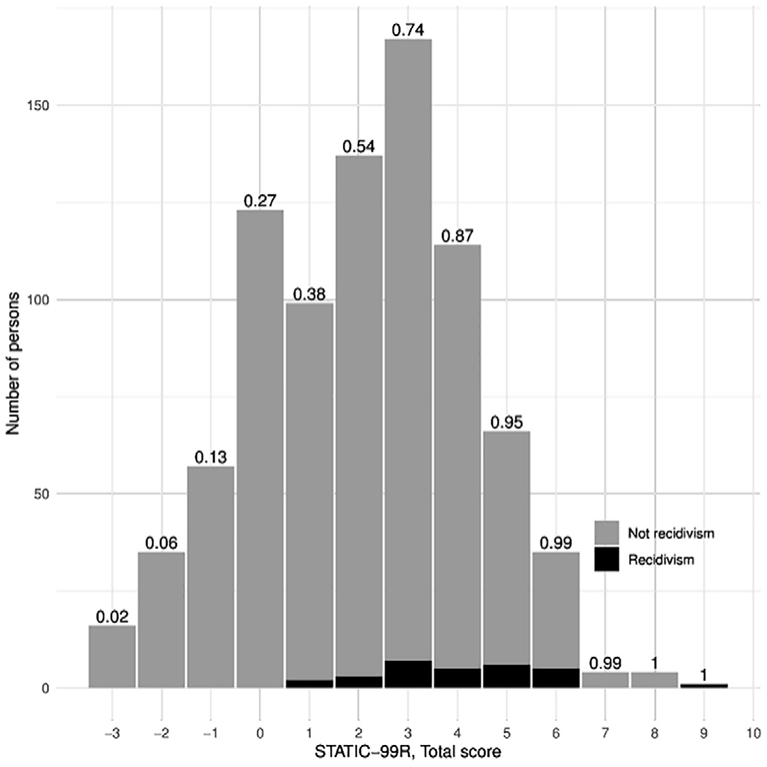
Histogram of Static-99R risk level scores by recidivism and not recidivism. *Note.* Figures on top of the bars are the cumulative proportions of Static-99R scores.

Average total score on the Static-99R was 2.06 (*SD* = 2.19, range = −3 to 9). After a mean follow-up time of 2,306 days, 9.9% (*n =* 85) of the sample recidivated to any type of offense, and 3.4% (*n* = 29) of the sample recidivated to a new sex offense. Static-99R risk level scores among the recidivists are presented in Figure 1, indicating higher recidivism among those scoring higher on Static-99R (see the appendix for the absolute numbers, as an elaboration to Figure 1).

Table 3 presents the regression estimates for sexual recidivism across the full observation period.

**Table 3.** Regression Coefficients From Logistic Regression and Proportional Hazard Models.

	Logistic	Cox proportional hazards
	Model 1	Model 2
Static-99R total score	0.48*** (0.10)	0.51*** (0.05)
Constant	−4.84*** (0.44)	
*N*	858	858
Log likelihood	−113.29	−589.14
AIC	230.58	1,180.28
AUC	0.76 (0.69, 0.84)	
Average cross-validated AUC	0.75	
Harrell’s C		0.80 (0.76, 0.84)

*Note.* AIC = Akaike information criterion; AUC = area under the curve.

* *p* < .05. **p < .01. ***p < .001.

The regression coefficient for the Static score is in the expected direction and statistically significant (β = .48, *SE* = .10, *p* < .001), with reasonably high predictive accuracy as AUC = .76 (fivefold, cross-validated AUC is similar: .75). Model 1 using only the Static-99R score is the more parsimonious and equally accurate.

The results from the proportional hazard model (model 2) is roughly similar to the logistic regression model, although with slightly higher predictive accuracy with Harrell’s C = .80.

**Figure 2.**
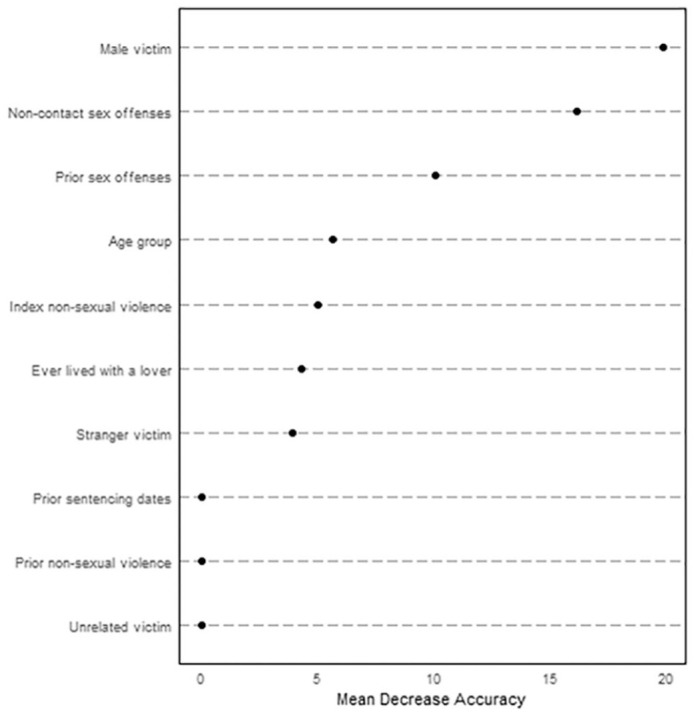
Relative importance plot from the random forest classification.

Finally, we used random forest classification algorithm, providing a standard calculation and visualization of the importance of each risk item for this model (Berk, 2016). Figure 2 visualizes to what extent each variable contributed to the overall predictive accuracy. Larger values indicate greater contribution to prediction holding the other variables constant.

Any male victim, any convictions for noncontact sexual offenses, as well as prior sex offenses, were clearly the most important predictors, followed by age group, index nonsexual violence, ever lived with a lover, and any stranger victim. The contribution from the remaining three variables was practically zero.

### Classification of Higher Risk

The Static-99R risk level classification identifies nominal risk levels IVa (total score 4–5) and IVb (total score 6+) as the higher risk groups, to be prioritized for further assessment. Using this classification, the cross-tabulation of predicted and observed recidivists is shown in Table 4.

**Table 4.** Comparison of Recidivism for High-Risk Group Classified by Static-99R and Random Forest.

Classification	Nonrecidivism (%)	Recidivism (%)	Total (*n* = 858)
Static-99R			
Not high-risk	622 (98.1)	12 (1.9)	634
High-risk	207 (92.4)	17 (7.6)	224
Accuracy			0.745
Random forest			
Classified as nonrecidivism	745 (98.8)	9 (1.2)	754
Classified as recidivism	84 (80.8)	20 (19.2)	104
Accuracy			0.892

For this classification, out of 224 persons classified as high-risk, only 17 recidivated (precision = 0.08), but giving an overall accuracy of 0.75, which was largely driven by the large share of correctly classified nonrecidivists. There were 17 times as many incorrectly classified recidivists (who turned out to be nonrecidivists) than there were recidivists incorrectly classified as nonrecidivists. If further intervention was offered to all predicted recidivists, that would amount to 26% of the sample. This would include 59% of the true recidivists, while only 8% of those included in the intervention would be actual recidivists.

Using the random forest algorithm for a similar cross-tabulation increased the accuracy to 0.89, largely driven by a higher number classified as nonrecidivists. For this classification, out of 104 persons classified as high-risk, 20 recidivated (precision = 0.19).

As with most screening instruments, there are costs associated with erroneous classifications. Lack of precision can lead to unnecessary interventions, but this must be weighed against the cost of not intervening against the true recidivists. One could say the classification using random forest would be a bit more cost effective in this study as the number of false positives was lower.

## Discussion

The results presented in this article support the relevance of Static-99R as a risk screen in a Norwegian context, providing similar results concerning predictive accuracy as previous studies using the same methods. The recidivism rate in our nonselected sample was 3.4% after a mean follow-up of 6.3 years after release from prison. Compared with the results presented in (Hanson et al., 2016), this places our Norwegian sample together with other routine samples using conviction (rather than charges) as recidivism criteria. The proportion being classified as below average risk (belonging to the two lowest risk levels) was 26.9% (*n* = 231), with an almost equal proportion (26.1%, *n* = 224) belonging to the two highest risk levels. The Hanson et al. (2016) results contained a slightly lower proportion in the lowest risk levels (22.6%) and slightly higher in the highest risk levels (32.9%).

According to Helmus and Thornton’s (2015) meta-analytic study, the single items of Static-99R contributed incrementally to the prediction of sexual recidivism, although the predictive accuracy of individual items varied across samples. In our study, the different methods used to investigate predictive accuracy all indicated that the five risk levels provided reasonable simplifications of the total risk score. The different methods suggested that some of the Static-99R items showed little predictive value. A similar finding was reported by Sjöstedt and Långström (2001) from their routine sample, although with different noncontributing items. As the absolute number of recidivists in both these studies is low, the results concerning the relative strength of individual items may be subject to instability and thus prohibit firm conclusions at this stage. It has been outside the scope of this article, but future studies might benefit from including measures such as fixed meta-analysis, directly comparing the results between different studies.

One of the obvious benefits of the Static-99R is that it is user friendly, not very time-consuming, and based on information that in most jurisdictions is easily available at prison intake. Each new correctional client’s recidivism risk may be calculated by hand by the practitioner. However, as seen from the results from the random forest algorithm, it is worth considering other methods of classifications using the same factors underlying Static-99R. One reason is the potential opportunity to improve accuracy. Although the regression analyses provided a high degree of accuracy (AUC = 0.76 and Harrell’s C = 0.80), this is largely driven by correct classification of nonrecidivists. All methods yielded a high number of incorrectly classified recidivists (persons identified as recidivist who did not actually recidivate), and a low number of incorrectly classified nonrecidivists (persons classified as nonrecidivists who actually recidivated). Thus, Static-99R seemed more accurate for rule-out decisions (correctly classifying nonrecidivists) than for rule-in decisions (correctly classifying recidivists), which was to be expected given the very low base-rate for recidivism. However, when resources are scarce, it can be important to apply methods that further optimize this cost-ratio balance. The number singled out for further assessment and possible intervention should preferably be low and accurate. That might not be achievable, but it might be more important to correctly classify true recidivists than inaccurately classifying as recidivist persons who do not go on to offend. Methods like random forest can be *tuned* to reflect such costs, and further work into getting a more desirable ratio of errors may be achieved. One current drawback of classification algorithms such as random forest is that they do not lend themselves to hand calculation, but require access to a computer. However, an app on a computer or even a mobile phone that allows entering the raw scores and submitting it to a central server should be easy enough to set up and no more complicated than some current calculations some use for Static-99R using a computer.

There are several ethical challenges involved in using actuarial screening instruments to sort inmates by risk levels (see Campbell, 2003). It may even be regarded as being at odds with the humanistic cornerstone of Norwegian Correctional Service. One may argue that standardized approaches based on shared characteristics among crime-specific subgroups of clients represent the opposite of an individualized approach to every correctional client, based on their unique characteristics as human beings. However, resorting to subjective evaluation based on professional judgment also has its perils and has repeatedly been outperformed by actuarial judgment (Duwe & Rocque, 2018). In line with previous studies, we argue that our findings support cautious application of Static-99R as an initial risk screen (Sjöstedt & Långström, 2001). As noted by Helmus and Babchishin (2016), “risk” is a concept “shrouded in uncertainty” (page 9), and risk assessment instruments, irrespective of their quality, are only to be regarded as aids in the complex task of trying to forecast future events.

### Strengths and Limitations

The fact that the current study is based on a routine sample without any type of selection is a considerable strength. The fact that the scoring of the Static-99R is based only on information that is routinely available in the Correctional Service at intake increases the potential of implementing the results, as no additional information needs to be gathered. However, there were some shortcomings. Information needed to score Item 2 was sometimes not available in this sample, but in such cases, this item should still be scored a “0” (zero) (as if the offender had lived with an intimate partner for 2 years) and will not be reported as missing. Thus, for scoring purposes, the shortcomings were mainly due to lack of information on previous charges (needed to score Item 5). The correctional client registry only systematically includes information on charges ending in a conviction. Charges dropped or ending in acquittal are not part of a person’s criminal record. Having relied mostly on convictions and to a far lesser degree been able to count charges may have led to an underestimation of risk of recidivism (see Sreenivasan et al., 2010).

In addition, our findings are based on persons sentenced to *prison* for a sexual crime and cannot be generalized to other sanctions administered by the correctional service (such as conditional sentences, community sentences, fines, etc.). Finally, we need to acknowledge the very low number of recidivists (*n* = 29) as a serious methodological shortcoming, as it makes the statistical models subject to unwanted instability. To avoid this problem, future studies should consider less conservative measures of recidivism, such as reoffense, or rearrests, what Andersen and Skardhamar (2015) call “front-end” measures (p. 7), rather than a “back-end” measure, as used in this study.

## Appendix

**Table:** Recidivists and Nonrecidivists in Absolute Numbers.

Total score	Nonrecidivism	Recidivism	Total *n*	Cumulative distribution (ecdf)
–3	16	0	16	0.0186
–2	35	0	35	0.0594
–1	57	0	57	0.126
0	123	0	123	0.269
1	97	2	99	0.385
2	134	3	137	0.544
3	160	7	167	0.739
4	109	5	114	0.872
5	60	6	66	0.949
6	30	5	35	0.990
7	4	0	4	0.994
8	4	0	4	0.999
9	0	1	1	1

## Notes

1. The Norwegian Penal Code was revised in 2005, moving sexual offenses to Chapter 26. We refer to the former version of the penal code, as the revised version was not officially implemented until October 2015.

2. Two main criteria define a Category B offense: (a) sexual behavior that is illegal, but the parties are consenting or no specific victim is involved and (b) indecency without a sexual motive (see Phenix et al., 2016a).
